# Indigenous Races of the Earth, or New Chapters of Ethnological Inquiry

**Published:** 1857-09

**Authors:** 


ART. II.—*Indigenous Races of the Earth; or New Chapters of Ethnological Inquiry;* including Monographs on Special Departments of Philology, Iconography, Cranioscopy, Palæontology, Pathology, Archæology, Comparative Geography, and Natural History. Contributed by ALFRED MAURY, Bibliothécaire de l'Institut de France, etc.; FRANCIS PULSZKY, of Lubocz and Cselfalva, and J. AITKEN MEIGS, M.D., Prof. of the Institutes of Medicine in the Philadelphia College of Medicine (with Communications from Prof. JOS. LEIDY, M.D., and Prof. L. AGASSIZ, LL.D.), Presenting Fresh Investigations, Documents, and Materials. By J. C. NOTT, M.D., Mobile, Ala., and GEO. R. GLIDDON, formerly U. S. Consul at Cairo, &c. Philadelphia: J. B. Lippincott & Co. London: Trübner & Co., 1857. 4to. pp. 656.

*Crania Britannica.* Delineations and Descriptions of the Skulls of the Early Inhabitants of the British Islands; together with Notices of their other Remains. By JOSEPH BARNARD DAVIS, M.R.C.S. Engl., F.S.A., etc., and JOHN THURNAM, M.D., F.S.A., etc. London, 1856. Decade I. 4to.

*Essai sur les Déformations Artificielles du Crane.* Par L. A. GOSSE, de Genève, Docteur en Médecine, &c. Paris, 1855. 8vo. pp. 159.

*Schædel, Hirn und Seele des Menschen und der Thiere nach Alter, Geschlecht und Race, dargestellt nach neuen Methoden und Untersuchungen von Emil Huschke.* Jena, 1854, 4to. pp. 194.

THIS is essentially an age of specialties. In every direction brain and muscle are individualizing their efforts. With each succeeding year the labors of the artisan are becoming more and more numerous, in consequence of the increasing wants engendered by the march of civilization; while in the vast field of science, restless and indefatigable pioneers are daily discovering and exploring new paths of scientific research before untrodden and unknown.

As the cell, at once the most simple and general expression of the organic world, in pursuing its developmental career, ever departs from its general characters, and through many transitionary or embryonic forms, seeks the specific, parental type and functions; or, as the individual slowly acquires, in the course of his growth, the traits which effectually distinguish him from other individuals of the same series; so, genera and species of animals and plants appear to advance in a more or less orderly manner to a certain point of development, through various phases foreseen and provided for by certain universal, immutable, and eternal laws. Herein we recognize a remarkable and instructive parallelism between the developmental careers respectively, of the cell, the individual, and the generic or specific group to which that individual belongs. Carefully examined, this developmental progression is seen to be an evolution of the special from the general; or as Von Baer expressed it, a gradual advancement from the homogeneous to the heterogeneous. In this advancement or evolution new peculiarities of structure or condition, indicating new capabilities and functional powers, gradually arise. A part of nature himself, man, also, falls within the scope and influence of this great law of evolution. Through many and varied changes, determined by fixed organic laws, every human being ultimately attains that mental and corporeal individuality which serves effectually to discriminate him from his fellows. If, now, we remember that a society is but an aggregation of individuals, and that the institutions of a society are but the collective acts of its members, we may at once suppose that the highest special culture of which any society or nation is capable, can be attained only in obedience to laws essentially the same as those controlling individual development. And such is, indeed, the case. Comte, the miscalled Bacon of the 19th century, has, in his positive, or rather dogmatic way, formuled the law of human progress, and his formula, though not very happily expressed, is, in essence, the same as that announced by Von Baer for the animate world at large. Comte expressly declares that the "progress of the individual mind is not only an illustration, but an indirect evidence of that of the general mind;" that the point of departure of the individual and the race are the same, and that, consequently, the phases of the mind of a man correspond to the epochs of the mind of the race.

Of the specializing tendencies of the age no better proof can be found than the manner in which Ethnology, the youngest of the Sciences, is already, while yet in its infancy, splitting up into various minor branches, all of which, however, tend to one great point,—the scientific explanation of the origin, affiliations, and destiny of the human races; in a word, the philosophy of Anthropology.

Ethnology is essentially the science of the age. It is at once the youngest and most aspiring of all the sciences. Treating of man in his physiological, psychical, and social relations, it necessarily embraces some of the loftiest problems which can occupy the mind. Its beginnings or elementary principles are the termini or results of other sciences, while its generalizations assume a magnitude unknown to those of any other inquiry. The data upon which these generalizations are based are freely drawn on the one hand, from the accounts which travellers have given us of the physical and mental characters of various races of men; their traditions and superstitions, their religious observances, their laws and customs, their habits of life, their vocabularies, &c.; and on the other, from the philologist and the zoologist, who, in the quietude of their closets, investigate respectively the grammatical structure and relations of various languages, and the anatomical and physiological details of structure, form, color, constitutional peculiarities, &c., and finally from the physical geographer and the climatologist, who have diligently recorded numerous observations upon the physical aspects and the climatic conditions of the various geographical areas occupied by the races of men.

Ethnology is, to a great extent, an American science. Upon American soil representatives from various parts of the globe have congregated themselves together during the last two hundred years. Upon American soil the primitive semi-civilized Toltecan man has acted out his allotted part, and become extinct; while in their turn, tribe after tribe of their successors, the barbarous autocthones, have faded away in obedience to a mysterious and irrevocable law, which has prepared the land as it were for the reception and accommodation of the Scandinavian, the German, the Sclavonian, the Kelt of Southern Europe, the follower of Mahomet, the disciple of Confucius, and the unhappy children of Africa, who with the strange, lozenge-faced Esquimaux of our polar coast line, and the fragmentary remains of the aboriginal Red Men, have assembled together, in obedience to laws of humanitarian progress and change, to work out the problem of human destiny. Nowhere else, therefore, on the face of the globe is so favorable and so extensive an opportunity offered for investigations of this character. In no other country can be found, in such close proximity, so many typical races of men, differing from each other in physical and mental characters. The remarkable contrasts which these races afford, the peculiar results which follow their contact with each other, and the profound mystery which shrouds the origin and early history of both the Toltecan family, and the

Barbarous tribes of our continent, are subjects which most strongly attract the attention of the philosophical observer. Moreover, to American scholars ethnology is indebted for much of its advancement. Among the distinguished names of its cultivators those of Morton, Gallatin, Squier, Davis, Nott, Bartlett, Van Amringe, and others, rank deservedly high.

As the beautiful golden ornament, before it assumes its pleasing form, is severely tested by fire in the crucible of the refiner, so the sciences generally, have succeeded in establishing their great and sublime truths, only after the most determined, and in some instances the most bigoted opposition. To this statement ethnology is destined to form no exception. Like astronomy in the time of Galileo, or palæontology in the days of Bernard Palissy, or geology in those of Hutton, ethnology is at the present time *la science militant.* The unfortunate controversy now going on in relation to the unity or diversity of human origin, and the permanence of human types, constitutes the furnace in which the youthful science is slowly undergoing a fiery ordeal. It is a curious fact that the earliest, and for a long time, the only attempt to bring the unity doctrine into disrepute, was made not by the naturalists, but by a theologian,—one Peyrerius; whose daring scholastic essay upon the Pre-Adamites, aroused the indignation of both Protestant and Catholic clergy, and was, by command of the Sorbonne, publicly burned in the Place de Grève. From that day (1655) to the present, this acrimonious discussion, originating thus in the domain of the theologian, and at first debated entirely upon theological grounds, has waxed stronger and stronger, and spread so widely, that it has come at length to embrace many eminent savans, who have either directly or indirectly espoused one or other side of the quarrel, and gradually arranged themselves into two great parties: the Unitarians and Diversitarians, or, to use the language of Mr. Gliddon, the Monogenists and Polygenists. In the first, we find the names of Prichard, Latham, Wiseman, Bunsen, Johnes, Serres, De Salle, Buchez, Klee, Bachman, &c.; in the second, those of Morton, Agassiz, Hamilton, Burke, Van Amringe, Maury, Knox, Caldwell, Jacquinot, Hombron, Virey, Desmoulins, Bory de St. Vincent, Blanchard, Lucas, Berard, Broc, Giebel, Klemm, Zeune, and many others. During the discussion, much talent and very diversified learning have been brought to bear upon the obscure points of the science, in some instances with the happiest results. Nevertheless, it is an acknowledged fact, and one much to be regretted, that in the course of the controversy ethnology has been made to assume a very offensive and suspicious attitude in the eyes of the people. Popular prejudices have been skilfully appealed to, so as greatly to disparage the science and its honest and laborious cultivators. The painful details of the controversy into which the late Dr. Morton was unwillingly forced, are fresh in the minds of many. The *odium theologicum* is not to be lightly encountered. The history of science—which is the history of intellectual progression—is replete with examples illustrative of this point. In view of these facts, therefore, we are of opinion that the legitimate work of the ethnologist, at present, is to accumulate facts, carefully sifting these, and separating the true from the doubtful and the false. In this way only can the first principles of his science be determined, and a distinct and settled nomenclature be agreed upon. We are well convinced that all this must be done before ethnology can venture, with any hope of success, to take the field in offensive armor.

Such are some of the thoughts which have arisen in our mind while turning over the leaves of the bulky quarto which stands first upon the list of works at the head of this article.

This work is in fact a *Thesaurus Ethnologicus*—a huge Encyclopædia, in which various special branches of ethnology are discussed with considerable learning, and at greater or less length, by different writers, all of whom are evidently well versed in their respective studies, while some of them are already celebrated in the scientific and medical world for their laborious and brilliant researches. Our readers must not suppose, however, that this work is the result of any concerted action on the part of its contributors. On the contrary, we learn from the *Prefatory Remarks* of Mr. Gliddon, that the various chapters composing it were written in the widely-separated cities of Paris, London, Cambridge, Philadelphia, and Mobile, without any communication between the writers, some of whom appear to be strangers to each other. The authorship is divided between France, Hungary, Switzerland, England, and America; between the author of the Poissons Fossiles, the Curator and Librarian of the Academy of Natural Sciences of Philadelphia, the Librarian of the French Imperial Institute, a Hungarian Exile, a Lieutenant in the United States Navy, a Southern Physician, and last, but not least, the indefatigable projector of the work himself—formerly American Consul to Egypt, now an important attaché of the Honduras Interoceanic Railway Company.

From this statement the reader will at once perceive how heterogeneous the sources, and how cosmopolitan the character of the volume under consideration.

The first three chapters are more or less didactic in their character. They constitute so many separate and distinct memoirs devoted respectively to the languages, arts, and skulls of the different races of men. The authors of these chapters, Messrs. Maury, Pulszky, and Meigs, confine themselves to a simple narration of facts, avoiding those *quæstiones vexatæ* to which we have already referred. Dr. Meigs, indeed, in the explanatory note prefixed to his contribution, expressly disclaims all reference to these questions.

"In the treatment of my subject," says he, "you will observe that I have confined myself chiefly to a simple statement of facts, carefully and designedly abstaining from the expression of any opinion upon the prematurely, and perhaps, in the present state of our knowledge, unwisely mooted questions of the origin and primitive affiliations of man. Not a little study and reflection incline me to the belief that long years of severe and earnest research are yet necessary before we can pronounce authoritatively upon these ultimate and perplexing problems of Ethnology."

The tone of the fourth chapter is argumentative. Dr. Nott here calmly, dispassionately, and with logical precision, argues the question of human origin, adducing in support of the diversity doctrine many important and striking facts relative to acclimation. The fifth and sixth chapters are emphatically controversial. The polemical powers of Mr. Gliddon, who is acknowledged to be the boldest, most determined, and aggressive of all the "polygenistic" writers,* are here exerted to their utmost.

A careful examination of this work clearly shows that there is by no means a perfect unanimity of opinion among the authors relative to the various facts and theories discussed therein. While the latter half of the volume contends positively and boldly for the theory of various origins for the various races of men, the first almost entirely avoids the expression of an opinion upon this point, even doubting, as we have just seen, the propriety of disturbing such ultimate questions at the present time, when the light necessary for their examination is so feeble and dim. We propose, therefore, to analyze each chapter in succession, and in as much detail as our limits will admit. Justice to both contributors and authors requires this procedure. Any attempt to characterize the whole work from the facts and arguments presented in a part, must necessarily be open to the charge of ignorance and want of discrimination.

In the Prefatory Remarks, we meet with three interesting letters addressed to Mr. Gliddon. The first, from Lieutenant Habersham, contains a curious account of a race of men inhabiting a portion of the Island of Formosa, and resembling in many respects the aborigines of North America, being like them, "of large stature, fine forms, copper-colored, with high cheek-bones, heavy jaws, coarse, black hair, reaching to the shoulders, and boasting no clothing save the maro, and a light cotton cloth over the shoulders."

The second communication is from the pen of Professor Agassiz. In it, notwithstanding the many erroneously-conceived attacks made upon his paper, published in Types of Mankind, and entitled, "Sketch of the Natural Provinces of the Animal World, and their relation to the Different Types of Man;" and notwithstanding the remarkable, and as it now appears, wholly unwarranted and injudicious apology for that paper which appeared in a recent number of the *Bibliotheca Sacra*, under a signature well known to the

* See the Canadian Journal of Industry, Science, and Art, for May, 1857, p. 209.

scientific world, Professor Agassiz reiterates, in still stronger terms, the opinions uttered by him in the work above mentioned. He says :

"So far as their geographical distribution upon the surface of the globe is concerned, the races of man follow the same laws which obtain in the circumscription of the natural provinces of the animal kingdom. Even if this fact stood isolated, it would show how intimately the plan of the animal creation is linked with that of mankind. But this is not all: there are other features occurring among animals, which require the most careful consideration, inasmuch as they bear precisely upon the question at issue, whether mankind originated from one stock, or from several stocks, or by nations. These features, well known to every zoologist, have led to as conflicting views respecting the unity or plurality of certain types of animals, as are prevailing respecting the unity or plurality of origin of the human races. The controversy which has been carried on among zoologists, upon this point, shows that the difficulties respecting the races of men are not peculiar to the question of man, but involve the investigation of the whole animal kingdom—though, strange as it may appear, they have always been considered without the least reference to one another.

"I need not extend my remarks beyond the class to which man himself belongs, in order to show how much light might be derived, for the study of the races, from a careful comparison of their peculiar characteristics with those of animals. The monkeys most nearly allied to man afford even the best examples. The orang-outans of Borneo, Java, and Sumatra, are considered by some of the most eminent zoologists as constituting only one single species. This is the opinion of Andreas Wagner, who, by universal consent, ranks as one of the highest authorities in questions relating to the natural history of mammalia; while Richard Owen, than whom no man, with the exception of our own Jeffreys Wyman, has studied more carefully the anthropoid monkeys, considers them as belonging to at least three distinct species. A comparison of the full and beautifully illustrated descriptions which Owen has published, of the skeleton and especially of the skulls of these species of orangs, with the descriptions and illustrations of the different races of man, to be found in almost every work on this subject, shows that the orangs differ from one another in the same manner as the races of man do; so much so, that, if these orangs are different species, the different races of men which inhabit the same countries, the Malays and the Negrillos, must be considered also as distinct species. This conclusion acquires still greater strength, if we extend the comparison to the long-armed monkeys, the Hylobates of the Sunda Islands and of the peninsulas of Malacca and Deckan, which extend over regions inhabited by the Telingans, the Malays, and the Negrillos; for there exists even a greater diversity of opinions among zoologists respecting the natural limits of the species of the genus Hylobates, than respecting those of the orangs, which constitute the genus Pithecus. I have already alluded, on another occasion, to the identity of color of the Malays and orangs : may we not now remember, also, a similar resemblance between some of the species of Hylobates with the Negrillos and Telingans ?"

The same difficulties occur in the classification of the monkeys of South America, especially the species of the genus Cebus which is exclusively

American. In fact, all over the American continent, both South and North, excepting only the Polar regions of the latter, the variation of species and the range of their geographical distribution are remarkably extensive. Everywhere species and varieties of animals, separated by feeble lines of demarcation, are multiplied in a striking manner, and spread over an unusually large area. To this peculiarly American feature, the numerous Indian tribes who claim to be autocthones, constitute no exception, as Morton has conclusively shown.

In the third letter, Prof. Leidy directs our attention to the palæontological aspects of Ethnography; expressing, in the course of his remarks, a strong suspicion that evidence will yet be discovered of the existence of man as early as the Glacial Period. It is to be regretted that Prof. Leidy has not given us a complete essay upon the relations of Palæontology to Ethnology, instead of his present brief but highly interesting and suggestive communication. The profound attention which he has so long bestowed upon the fossil world, amply fits him for such an undertaking.

Thus much for the *Introduction* to "Indigenous Races." In Chapter I, the work fairly opens with an elaborate essay upon the distribution and classification of languages; their relations to the geographical distribution of races; and the inductions which may be drawn from these relations. M. Alfred Maury, the author of this essay, has already, through his numerous and learned philological and ethnological writings, achieved for himself an enviable transatlantic reputation. This side of the Atlantic, his scientific labors have not, as yet, received the attention which they deserve.

Unlike most of his cotemporary philologues, Maury recognizes the organic character of language, and discusses it in accordance with the well-tried methods of the naturalist. In so doing he renders philology a true auxiliary in the study of ethnology, and deprives it of much of the uncertainty with which it has hitherto been surrounded. Notwithstanding the importance claimed for philology by Prichard, Latham, and others, we have always been inclined to regard it as a science of dubious powers and results. However, in the hands of Maury, Pott, Renan, and others, constituting a new and vigorous school of naturalistic philology, the science is beginning to assume a novel and imposing aspect. With a masterly hand our author sets forth the causes and laws of the development and transformations of language. In these transformations language is seen to be not a dead product, but an animate and ever-living being, as Wilhelm Von Humboldt has expressed it,—a being which cannot remain stationary, but on the contrary develops itself, reaches maturity, sinks into old age and decrepitude, and at length dies. In this development of a language from its condition as a primitive, phonetic radical, rendering the idea or the sensation in all its simplicity and its generality, to the attainment of the most complex and artificial forms, we have another proof of the law already alluded to—the law of development from the general to the special. These complex and artificial forms vary with different races, and yet the fundamental mechanism is found to be the same in all languages, since that mechanism is dependent partly upon the nature of the mind, which is essentially the same for all mankind, and partly upon external nature, which everywhere repeats itself, and is everywhere the expression of an infinite diversity in unity. Languages undergo alteration in consequence of a continued development within themselves, and by their contact with other languages possessing different idioms. A language abounding in synthetical expressions, and compound phrases, when brought in contact with a more analytical tongue, generally loses some of its compound forms, adopting instead the simpler forms of the less advanced nations. However, we are assured by Maury, that the crossing of languages, like that of races, has really not been very deep, but that a language invaded by one more logical in its construction, either suffers merely slight or superficial alterations, or disappears entirely, without bequeathing to the tongue which follows it any inheritance but that of a few words. It would thus appear that, during their existence, languages are remarkably tenacious of their peculiar grammatical forms. This fact is admirably illustrated in the case of the Greek and German, which, though separated from the parental Sanscrit stem for more than three thousand years, have nevertheless preserved a common stock of grammar.

"If the grammatical dispossession of a language could have been wrought gradually, one ought to find some mixed phrases at the living period of those tongues that have been driven out by others. Now, such is not the case. The *Basque*, for example, foreign in origin both to French and Spanish, has indeed been altered through the adoption of a few words and a few locutions borrowed from these languages, by which it is surrounded, and, as it were, invested; but it evermore clings to the basis of its structure, the vital principle of its organism; and a Franco-Basque, or a Basco-Spanish, is not spoken, nowhere has ever been spoken. Modern Greek has appropriated many words from Turkish, no less than from Italian, as well as some expressions of both tongues; but its entire construction remains fundamentally Hellenic, notwithstanding that it belongs to the analytical period, and that the ancient Greek was still emerging from the synthetic. Again, the Persian, which is so imbued with Arabic words that writers of this language often intercalate sentences wholly Arabic in their discourses, remains, nevertheless, completely Indo-Germanic as concerns its grammar. But we have not seen that this tongue has ever associated the Persian declension with the Arabic conjugation, or yoked the Persian prepositions to Semitic affixes and suffixes. Finally, the Osmànlee Turkish, besides incorporating words of every language with which the Turks have been in contact for more than a thousand years, has purloined all its scientific nomenclature from the Arabs, most of its polite diplomatic phrases from the Persians; but, whilst fusing Semitic as well as Indo-European exotic words into its *copia verborum*, the radical structure of its so-called Tartarian [or, Turanian] grammar, no less than its original vocabulary, is still so tenaciously preserved, that a coarse Siberian *Yakut* can even now, after ages of ancestral separation, communicate his simple ideas to the intelligence of a Constantinopolitan Turko-Sybarite."

Such facts, we may observe *en passant*, militate strongly against the hypothesis of a unique primitive language, whence all others have issued.

Notwithstanding the perdurable character of languages, we are by no means justified in relying upon them, to the exclusion of anatomy and physiology, in our attempts to elucidate and solve the obscure and complex problems of Ethnology. Latham, it is true regards physical characters, as less important than philological, moral ones, while the Professor of Linguistics, at the University of Halle,* "claims for languages an ethnological character, suited to the classification of races, not less certain than the physical type and corporeal forms." He regards the idiom as a criterion even more certain perhaps than the physical constitution. Maury and Bodichon assign a higher value to physiology. The former, in an article published in the *Revue des Deux Mondes* for September, 1855, expressly declares that we are indebted to the monuments of Egypt for the demonstration of the two great historical laws that dominate over all the annals of that land, viz.: the permanence of races, and the constant mobility of tongues, beliefs, and arts—two truths which are precisely the inverse of what had been, for a long time, admitted.† Professor Agassiz is disposed to call in question the value and reliability of philological evidence, as proving genetic derivation, while Dr. Meigs, in his present contribution to craniographic science, combats the opinions of Latham, condensing in one paragraph the most cogent objections to the view of this eminent philologist.

"When we contemplate," says he, "the mutability and destructibility of languages, as abundantly exemplified in the obliteration of the Etruscan dialect by the Roman-Latin; the Celtiberian and Turdetan by the Latin and Spanish; the Syriac by Arabic; Celtic by the Latin and French; the Celtic of Britain by the Saxon and English; the Pelhevi and Zend by the Persian, and the Mauritanian by Arabic; when we reflect how the Epirotes and Siculi changed their language, without conquest or colonization, into Greek, and how the ancient Pelasgi, all the primitive inhabitants of the Peloponnesus, and many of those of Arcadia and Attica, abandoned their own language and adopted that of the Hellenes; when we behold the Negroes of St. Domingo speaking the French tongue, the Bashkirs, of Finnish origin, speaking Turkish; and when, finally, as one instance of another and significant class of facts, we call to mind how the Carelians, in consequence of

* M. A. F. Pott. Die Ungleichheit menschlicher Rassen haupsächlich vom Sprachwissenschaftlichen Standpunkte. 1856.

† See also, his recent work entitled "La Terre et L'Homme," Paris, 1857, chap. viii,—Des Langues et de leur Distribution Géographique.

certain linguistic analogies, have been classed with the Finns, though descended from an entirely different race, who, at an early period, overran the region about Lake Ladoga, we are 'disposed to believe with Humboldt,'—I am using the words of Morton—'that we shall never be able to trace the affiliation of nations by a mere comparison of languages; for this, after all, is but one of many clues by which that great problem is to be solved.' Surely anatomy and physiology—those handmaids of the zoologist—are more powerful, and, in the very nature of things, better adapted to settle the question of the unity of man, to determine whether the human family is composed of several species, or of but one species comprising many varieties. Surely the human skeleton is more enduring and less mutable than the oldest language. Instances are not wanting, as we have seen above, of a nation forgetting its own language in its admiration for the more perfect speech of another people. But, as far as I am aware, not a solitary instance can be adduced of a nation, *genealogically pure*, entirely changing its physical characters for those of another."

In the third section of his paper, Maury proceeds to show how the languages of Europe belong to a great family, which, at an early hour, divided itself into many branches, of whose common ancestor we are ignorant, but of which we encounter in the Sanscrit the chief of one of the most ancient collateral lines; and also how a comparison of the languages of Europe has caused them to be grouped into four great classes, representing, as it were, so many sisters sprung from the same mother, but sisters who have not been called to an equality of partition. These classes or families are the Pelasgic, Shlavic, Indo-Germanic, and Celtic. The latter is the farthest removed from the Sanscritic mother tongue, both in geographical position and grammatical construction. The resemblance between the languages of Europe, affiliated as these are, in common, with idioms which, at a very remote humanitarian period, spread from the shores of the Caspian to the banks of the Ganges, is regarded by Maury as a weighty argument in favor of the Asiatic origin of most of the European races. Prior to the migration of ancient tribes from Asia, however, the Iberes or *Yonderers* appear to have occupied the soil from the Alps to the western borders of Spain. Now, the Basque or Euskarian tongue, spoken by these Iberians, in no way resembles the Indo-European idioms, as was formerly shown by M. Von Humboldt, and still more recently by M. Boudard of Beziers. It is, in fact, a polysynthetic language, which, in its organic structure, resembles the native languages of America, and indicates a very primitive intellectual state of the people who occupied Western Europe, previously to the arrival of the Indo-Europeans. But it appears, also, that the peculiarities of the Basque, its agglutination, its curious postpositions representing cases, its inability to simplify the relations of words to each other, notwithstanding its apparatus of forms, &c., all appear again among the Tartars of Central Asia, and among the Finns and the tribes generally which are scattered throughout

Northern Russia, from Finnmark, even to the extremity of the Kamtschatkan peninsula. Linguistically, the Tartar race attaches itself to the Finnish or Ugrian through the Tungusian type. The Mongolic, Mantchurian, Ugrian, and Turkish tongues, are allied to the Tchudic or Finnish, a fact which explains the resemblance between the Turkish and Hungarian languages. On the other hand, the languages spoken by the tribes of Siberia, and those employed by the races of Central Asia, differ from each other in a striking manner. We shall presently see that these conclusions of the philologist are in many respects confirmed by the craniological investigations of Dr. Meigs, recorded in the third chapter of this volume.

Iconographic Researches on Human Races and their Art constitute the subject-matter of Chapter II. This chapter is contributed by Francis Pulszky, the same gentleman, we believe, who accompanied the celebrated Kossuth, during his oratorical peregrinations through our country.

Blumenbach, the father of craniographic science, was among the earliest, if not, indeed, the first, to recognize the importance of Iconography as an auxiliary in the elucidation of the great problems of ethnology. We have in our possession a tract, published at Göttingen, in 1808, in which he distinctly alludes to it.* His unpublished lecture, delivered in Göttingen, March 19th, 1823,† and entitled "*De veterum artificium anatomicæ peritiæ laude limitanda, celebranda vero eorum in charactere gentilitio exprimendo accuratione*," was simply a recognition of the fact and nothing more. He appears never to have attempted a systematic elaboration of this idea. Prichard, in the second volume of his *Researches*, very briefly alludes to the remains of Egyptian painting and sculpture, as affording some aid to his favorite science. It was not until the publication of the *Crania Ægyptiaca* of Morton, however, that this subject was distinctly and powerfully made available. Courtet de Lisle, in his valuable and already scarce *Tableau Ethnographique du Genre Humaine*, employs the same method with discrimination and success. In *Types of Mankind* the subject was greatly enlarged upon by Messrs. Nott and Gliddon, and now in the present volume we find it receiving, at the hands of Mr. Pulszky, the attention which its importance deserves. Conceived in the highest style of critical analysis, the remarks of this gentleman are well calculated to arrest the notice of the reader. His essay we regard as the most excellent, and for its limits, the most complete attempt, with which we are acquainted, at explaining certain ethnological propositions, such as the constancy of natural types, &c., by comparing together authentic representations drawn from Egyptian, Assyrian, Persian, Greek, Etruscan, Roman, Hindoo, Chinese, and American monu-

* Specimen Historiæ Naturalis Antiquæ Artis Operibus illustratæ eaque vicissim illustrantis.

† Noticed in the Göttingen Gelehrte Anzeigen for that year.

ments. National art, viewed in the manner here indicated, becomes an important avenue, also, to the study of intellectual philosophy; for, like laws, customs, manners, and other societary institutions, it is in fact the objective or external manifestation of the power and genius of the national or tribal mind. Inequalities in the artistic power of different nations are in fact indications of some of those mental differences, which appear to be as old as the races themselves.

"Whilst it is certain that art has never flourished but among the progressive races, we shall find that nations to whom we are indebted for some of the most important discoveries, and to the highest truths revealed to mankind, are altogether deficient in art,—as, for instance, the Shemites without exception; that others, although wielding the most extensive political power, such as the Romans of old, the Scandinavian Northmen, the Anglo-Saxons, the Sclavonic races, never attained a high development of painting and sculpture, and were surpassed by the Greeks of yore, and by the Italians and Spaniards, the Germans and Dutch. History teaches us that eminence in painting and sculpture is not the result of either high mental culture or political power, and that it does not always accompany the refinement and wealth of nations. We find it growing out of a peculiar disposition of some nations, predestined as it were for art; whilst other races, living under the same social, climatic, and political conditions, never rise artistically to represent the outward world in colors or in plastic forms. And again, among the artistical nations we meet with the most remarkable differences in treating the same subjects. Some strive for the most scrupulous *reproduction* of nature, and cling to faithful imitation; others are creative, embellishing whatever they touch: some show a deep understanding and love of nature; others concentrate their power exclusively on the representation of the human body: some excel by the brilliancy and harmony of their coloring; others charm by their correctness in plastical forms: but all of them express their nationality, their peculiar relation to God, nature, and mankind, throughout their works. Therefore, even an inexperienced eye catches the difference between Egyptian and Assyrian, Indian and Chinese, Greek and Etruscan, Italian and German, French and Spanish, art: and the artistically-educated student feels no difficulty in discriminating the minute distinctions of schools, in each national art; and generally discovers any attempt at forging pictures and statues."

From the languages and arts of the various races of men, we pass on to the consideration of their cranial forms, the theme to which is devoted the whole of Chapter III. In this long and important chapter, contributed by Dr. J. Aitken Meigs, we find ourselves upon strictly scientific grounds, Ethnology being here investigated from the stand-point and after the methods of the naturalist. Dr. Meigs presents us with a comprehensive panoramic survey of the diversified cranial characteristics of the human family; a survey in which he assures us that he has had no theory to establish or defend, and has therefore carefully and impartially presented the facts as he has found them, for the most part indelibly traced upon the specimens in the celebrated Mortonian collection; a collection which he has taken much pains to arrange and classify, and of which he has recently given to the world a complete and systematic catalogue.* Dr. Meigs' contribution, we may say in passing, is marked by considerable industry and research, as shown by his frequent references to and quotations from the more important of the numerous works which constitute the literature of craniographic science.

With Buffon and Linnæus the study of man, as a science, fairly commences. Glimpses of this science may be caught in the writings of Thucydides, Herodotus, Hippocrates, Aristotle, Plato, Sallust, Cæsar, and Tacitus. Of these the Germania of Tacitus is undoubtedly the closest approximation to proper Ethnology that antiquity has furnished. Both Linnæus and Buffon directed their attention to external characters chiefly,—such as color of skin, hair, &c.; cranial characters, and the bony system they entirely overlooked, and in doing so failed to recognize the zoological relations of man. Nor was it until the publication of the *Règne Animal* of Cuvier, that these relations were clearly perceived. In 1764, Daubenton published his observations on the basis cranii and the variations in the position of the foramen magnum occipitis in different races of men. The cranium was next studied in profile by Camper, who attempted to compare national crania by their facial angles. Still more recently (1785), Sœmmerring applied the occipito-frontal arch, the horizontal periphery, and longitudinal and transverse diameters of the cranium, to demonstrate the differences between the heads of Europeans and negroes. At length, during the latter part of the 18th and the early part of the 19th centuries, appeared the *Decades Craniorum* of Blumenbach, who is justly regarded as the founder of the Comparative Craniography of the Races of Men. Passing over the theses of Gibson,† Crull,‡ and others, published about this time, we come at length to those magnificent works, the *Crania Americana* and *Crania Ægyptiaca* of Morton, the illustrious leader and founder of the American School of Ethnology. Ever since the publication of these works the scientific claims of Craniography have been generally acknowledged. The next important contribution to this science is the *Anthropologie* (the Crania Polynesiana, as it might not inaptly be called), of Dumoutier and Blanchard. In no work that we are aware of are the skull-forms of the Polynesian Islanders so well described as in the text of Blanchard, or so well illustrated as in the magnificent Atlas of Dumoutier. From the English press has just been issued the First Decade of the *Crania Britannica* of Messrs. Davis and Thurnam, at once the most

* Noticed in the last number of this Journal.

† Dis. Phys. Inaug. de Forma Ossium Gentilitia, Edinburgi, 1809.

‡ Diss. Anthropologico-Medica de Cranio, ejusque ad Faciem Ratione, Groningæ, 1810.

recent and only elaborate addition to Craniography that has yet appeared in Great Britain. From an attentive perusal of its pages, and careful inspection of its most artistically executed lithographs of the skulls of the ancient Britons, we can safely say that it will prove an invaluable addition to the library of the student of Human Natural History. The monograph of Dr. Gosse is equally invaluable to the Ethnologist, constituting as it does, the most complete essay that has yet appeared, upon the numerous deformations of the cranium, both natural and artificial, which are encountered among the various races of men. A specialty within a specialty, so to speak, it well illustrates the rapid manner in which Craniography, yet in its infancy, and with most of its fundamental principles yet to establish, is dividing and subdividing into its detailed and special sections, while it furnishes another proof of how the special is ever evolving itself from the general.

Germany has furnished us with quite a number of excellent memoirs and monographs connected with craniology. The most recent and most important of these, as far as we are aware, is the laborious treatise of Prof. Huschke, of Jena, the admirable result of nine years of study and reflection. With the exception of an excellent paper on cranial measurements, contributed by Mr. J. S. Philips to the second part of Schoolcraft's ponderous quartos on the Aboriginal Races of America, and the chapter in Types of Mankind on the Comparative Anatomy of Races, from the pen of Dr. Nott, of Mobile, nothing has been done for Craniology on this side of the Atlantic, since the death of Dr. Morton, until the publication of the lengthy essay of Dr. Meigs on the national forms of skulls.

In the first section of this essay, Dr. Meigs, after pointing out the differences between the human cranium and that of the lower animals, assures us,

> "That among the different tribes of monkeys, as among the various races of men, there are numerous types or forms of skull; that for each of these natural groups, there is a gradation of cranial forms; that the greatest resemblances between the two groups—resemblances indicating the existence of a transitionary or connecting link as a part of nature's plan—are to be sought for in or between the lower types of each, and not between the lowest man and highest monkey, as is generally supposed; that the undeveloped crania of the Chimpanzee, Orang, and other higher types of monkeys, more closely resemble the human form than when fully evolved; that for each of the lower human types of skull, there appears to exist among the monkeys a rude representative, which seems remotely and imperfectly to anticipate the typical idea of the former, and to bear to it a certain ill-defined relation; and, lastly, that the best-formed human skull stands immensely removed from the most perfectly elaborated monkey cranium."

Some remarks on the scope, importance, and general character of Craniography, are followed by a concise history of its origin, progress and present condition. Notice is also taken of the Mortonian Collection, now permanently deposited in the Museum of the Academy, and numbering nearly 1100 skulls. Of this vast collection Dr. Meigs has availed himself freely. In the second section, typical forms of crania, their relative antiquity and permanence, true nature and value of the characters by which species and types are established, the relative value of physiology and philology as ethnological instruments of research, the methods of discriminating between typical or race-forms of crania, and those modifications of shape produced to a certain extent by age, sex, development, intermixture of races, artificial deformations, &c., the transitionary forms through which the brain and cranium pass before they attain their full and normal development, the nature of cranial sutures, and their influence upon race-forms of the cranium, the action exerted by the muscles upon these forms, and finally the power exerted by civilization in modifying these forms, are all discussed at greater or less length, according to their comparative importance.

In the third section, after a critical analysis of some of the more important attempts at cranial classification, such as those of Blumenbach, Retzius, Zeune, Prichard, and others, Dr. Meigs proceeds to pass before us in review the cranial forms of the various races of men. And here it is that the full value of the Mortonian Collection becomes apparent, for without such a collection to refer to, this portion of the paper under consideration could scarcely have been written.

According to Dr. Meigs, with very slight and insignificant variations, the pyramidal type of skull prevails along the whole American coast north of the 60th parallel, and from the Atlantic Ocean to Bhering's Straits, ranging through 140° of longitude, or over a tract of some 3500 miles. It is encountered also amidst the snowy islands of the Polar Ocean, and upon the frozen *tundras* of Siberia. This type is characterized by a long, narrow, and keel-like calvaria, while the face, in consequence of the projection of the malar bones, has a peculiar lozenge or pyramidal shape. It embraces the Eskimos of America, the Tchuktchi of Bhering's Straits, the Samoiedes of Siberia, and probably the Koriaks and Aleoutians.

The Kamtschatkans are regarded as a divergent or modified variety of the Arctic type. In the form of their skull, as well as by their language, the Yakutes of the Lena are affiliated to the Turkish family. With regard to the Laplanders we find the following paragraphs on pages 268 and 288 :

"A critical examination of three Laplander crania, and two casts, contained in the collection of Dr. Morton, and a comparison of these with a Kalmuck head and a number of Finnic skulls, convince me that the Laplander cranium should be regarded as a sub-typical form, occupying the transitionary place between the pyramidal type of the true Hyperboreans on the one hand, and the globular-headed and square-faced Mongol on the other. Just as upon the shores of Eastern Asia, we behold the Arctic form passing through the Kamtschatkan and the Southern Tungusian into the Central Asiatic type, so in the western part of the great Asio-European continent, we behold a similar transition through the Lapponic into the Tchudic and Scandinavian types—the most northern of the European.

"It will thus be seen that the cranial type of the Laplander belongs to a lower order than that of the Finn, and that the former race falls properly within the limits of the Arctic form, while the latter leans decidedly towards the Indo-Germanic type, finding its relation to the latter through the Sclavonian rather than the true Scandinavian types. But inferiority of form is to some extent a natural indication of priority of existence. We are thus led from cranial investigations alone to recognize the Lapps as the autocthones of Northwestern Europe, who at a very remote period have been overlaid by the encroaching Finn."

In Central Asia, a globular conformation of the skull takes the place of the long, pyramidal type of the North. To this globular-headed and square-faced type belong the Kalmucks, Kalkas, Bouriats, Kirghiz, Don Cossacks, Usbecks, Nojai Tartars, and other tribes inhabiting the heart of Asia. The skull of the Turk appears to be of a peculiar form, standing midway between that of the European and Mongol, and graduating into the European type through the skulls of the Osmanli Turks and the Tartars of the Kasan. The Turkish, Mongolian, and Tungusian crania bear to each other a general family resemblance. This physical fact to a certain extent confirms the opinion of Klaproth, that these three great nomadic races are philologically related.

"Indeed, the Tungusian tribes seem to connect the Chinese with the frozen North; for, in a modified degree, the same differences which separate the true Hyperborean from the typical Mongol, also separate the Chinese from the latter. In other words, the Chinese nation, in the form of their heads, resembles the great Inuit family more than the Mongolian. This opinion is based upon the critical examination of eleven Chinese skulls, obtained from various sources, and now comprised in the Mortonian Collection."

Of the tribes of the Trans-Gangetic or Indo-Chinese and Hindostanic Peninsulas, Dr. Meigs thus speaks:

"The Indo-Chinese nations, including the Mantchurian Tungus, or those south of the Alden, should be regarded as a distinct but closely allied type, a type bearing certain resemblances to the pyramidal form on the one hand, and the globular on the other, but positively separated from these two by certain slight but apparently constant differences. The Koreans exhibit the same type. * * * * *

"The above descriptions evidently lead to the recognition of several varieties or sub-types of cranial form in the Indo-Chinese Peninsula, some of which are more or less related to the predominating type of Central Asia, while others approximate the Malayan, and through these the Polynesian forms. Indo-China may therefore be regarded as the transitionary or debatable ground between Asia and Polynesia. * * * * *

"The Mortonian Collection contains in all forty-three crania of the Indostanic Race. Among these skulls, at least two types can be distinguished. 1st. The fair-skinned Ayras, a conquering race, speaking a Sanscrit dialect, and occupying Ayra-Varta, which extends from the Vindya to the Himalaya Mountains, and from the Bay of Bengal to the Indian Ocean, and comprises the Mahrattas, and other once powerful tribes, who have so boldly and obstinately resisted the English arms. 2d. The Bengalee, represented by thirty-five skulls. Dr. Morton considers these small-statured, feeble-minded, and timid people as an aboriginal race upon whom a foreign language has been imposed."

Asia, craniologically, graduates into Europe, not only through the Osmanlis and Khazan Tartars, but also through the races constituting the widely-spread Finnic or Tchudic family, which, at an epoch antedating the earliest records, occupied the country extending from Norway to the Yennisei, north of the 55th degree of latitude in Asia, and the 60th in Europe. Through the Affghan skull the Indostanic blends with the Semitic form. Some twenty-five pages are devoted to the consideration of the crania of Northern, Central, and Western Europe, with the following results:

"It will thus be seen, from the foregoing observations on the crania of the races of Northern, Central, and Western Europe, that we must distinguish for these regions several distinct cranial types,—a Lapponic, a Finnic, a Norwegian, a Swedish, a Cimbric, a Germanic, an Anglo-Saxon, a Keltic, &c.; that the modern Finn represents, in all probability, the ancient Tchudic or Scythic tribes; that the Norwegian and Swedish are varieties of the same type; that the Germanic form is intermediate between the Finn and Swede; that the Anglo-Saxon skull is allied to the Swedish, its facial portion bearing, to some extent, the Finnic stamp; that the Cimbric type is very ancient (more ancient, perhaps, than any of the forms just enumerated, except the Lapponic), resembles the kumbe-kephalic, and represents a primitive humanitarian epoch; that the Keltic type, if indeed any such exists, should be regarded as a variety of the Cimbric—a low and early form; and lastly, that the various types of skull to a certain extent approach, represent, and blend with each other, in obedience to the great, and, as yet, not properly understood law of gradation, which seems to pervade and harmonize all natural forms, and in consequence, also, of the amalgamations which, within certain limits, must have accompanied the successive occupancy of this region by the races of men under consideration."

But we must hurry on. Our limits will not permit us to dwell any longer upon the various physical peculiarities and cranial forms presented by the various races of men. Let it suffice, therefore, to say that the next forty-two pages of the paper under discussion, are occupied with carefully-drawn descriptions of the physical, and especially the cranial, characters of Sclavonians, Hungarians, Dacians, Wallachians, Bulgarians, Albanians, Greeks, Romans, Etruscans, Phœnicians, Circassians, Armenians, Persians, Affghans, Arabians, Assyrians, Hebrews, Egyptians, Fellahs, Copts, numerous African and American tribes, the races of Polynesia, Malays, Australians, &c. &c. The paper concludes with a cautious and impartial attempt to indicate the conclusions which appear fairly to flow from the numerous facts and arguments brought forward by the author.

"Aware of the extreme caution necessary in arriving at conclusions in so grave a study as that which has just occupied our attention through so many pages, and knowing that every erroneous inference must either directly or indirectly retard the advancement of Ethnography, I have preferred, occasionally, to suggest what appeared to me a legitimate induction, rather than to pronounce positively and authoritatively upon the facts presented. In the same cautious manner, the following propositions are placed before the reader, as more or less clearly derivable from the foregoing facts and arguments.

"1. That cranial characters constitute an enduring, natural, and therefore strictly reliable basis upon which to establish a true classification of the races of men.

"2. That the value of such characters is determined by their constancy, rather than by their magnitude.

'3. That these characters constitute, in the aggregate, typical forms of crania.

"4. That historical and monumental records, and the remains found in ossuaries, mounds, &c., indicate a remarkable persistence of these forms.

"5. That this persistence through time, as viewed from a zoological stand-point, renders it difficult, if indeed possible, to assign to the leading cranial types any other than specific values.

"6. That, in the present state of our knowledge, however, we are by no means certain that such types were primitively distinct. The historical period is too short to determine the question of *original* unity or diversity of cranial forms. Moreover, this question loses its importance in the presence of a still higher one,—the original unity or diversity of *all* organic forms.

"7. That diversity of cranial types does not *necessarily* imply diversity of origin. Neither do strong resemblances between such types infallibly indicate a common parentage. Such resemblances merely express similarity of position in the human series.

"8. That each well-marked cranial type admits of certain variations in its individual characters, which variations constitute divergent forms.

"9. That these divergent forms must not be confounded with hybrid types. Both, it is true, are produced by modifications in the mode of action of the developing principle; in the former, however, these modifications depend upon climatic conditions, in the latter they result from race-amalgamation.

"10. That reasons exist for considering some, at least, of the so-called artifical deformations as strictly natural types, representing very early humanitarian epochs.

"11. That a regular system of gradation seems to underlie and harmonize the various cranial forms of the human family.

"12. That these forms appear to be pre-represented or anticipated in the various types of skull exhibited by different genera and species of monkeys.

"13. That if we regard artificial deformations as the forced imitations of once natural types, and upon this ground admit them in our systems of classification, as some writers have done, then the perplexing gaps which seem to break the animal chain by disparting man and monkeys—the group which stands nearest to man—will, to a certain extent, be filled intelligibly.

"14. That typical forms of crania increase in number as we go from the poles to the equator.

"15. That the lower forms are found in the regions of excessive cold and excessive heat; the higher occupying the middle temperate region.

"16. That cranial forms are inseparably connected with the physics of the globe."

In Chapter IV, Dr. Nott, in his usual clear and forcible manner, investigates the subject of Acclimation; or, the comparative influence of climate, endemic and epidemic diseases, on the races of men.

Dr. Nott commences by defining climate, and dividing it into two great varieties, physical and medical. After briefly alluding to the kind and degree of influence exerted by climate, upon the Faunæ and Floræ of particular geographical regions, he proceeds to speak of man in relation to the climatic conditions which surround him, contending that in respect to these conditions each species of man must be made a separate study. "When and where," he asks, "have the people of the North become habituated to the climate of the Tropics, or those of the Tropics been able to live in the North? We have no records to show that a race of one extreme has ever been acclimated to the opposite." Only in the comparative physiology of the races of men, a great book yet to be written, can such questions and propositions find their true place and value? In the meantime the able work of Boudin,* just issued from the French press, will furnish the reader with much valuable information on these interesting topics.

In considering the climate of the tropics and adjacent regions, Dr. Nott divides medical, into non-malarial and malarial climates. Under the head of malarial climate, facts are brought forward to prove that the races of men are influenced differently, not only by the temperature of various latitudes, but by morbific agents, which, to a certain extent, are independent of mere temperature, such as the causes of marsh or yellow and typhoid fevers, cholera, plague, &c.

The subject of acclimation of the white races in tropical climates, next receives attention, and is treated in the most interesting manner. From the extensive tables of Moreau de Jonnès, from various works devoted to the consideration of the influence of tropical climates, and from the valuable statistical reports of the British Army Surgeons, Dr. Nott draws a series of facts which seem conclusively to show that the white races of men cannot be acclimated in foreign tropical regions. The loss of life which has invariably followed the attempt has been terribly great. From his own observations and from those of many physicians, Dr. Nott assures us that among the in-

* Traité de Géographie et de Statistique Médicales, 2 vols. 8vo.

habitants of our Southern States there is no acclimation against the endemic fevers of the rural districts, and that those who live from generation to generation in malarial districts, become thoroughly poisoned, and exhibit the thousand protean forms of disease which spring from this insidious poison. He thinks that the so-called acclimation of negroes has been overrated, and that they never, as far as his observation extends, become proof against intermittents and their sequelæ. The malarial climates of the old world are next examined. In Italy the native population appears to be no better protected against the miasmatic poison, than it was two thousand years ago. The writings of the distinguished military physician, M. le Docteur Boudin, abound with statistics which show that in Algeria, the climate tends to the extermination of Europeans, for the yearly mortality, among the latter, instead of diminishing under acclimation has steadily increased. In view of all these facts it would seem indeed

"That certain races cannot become assimilated to certain climates, though they may to other climates far removed from their original birth-place. The British soldiers and civilians enjoy even better health at the Cape Colony than in Great Britain; while the negro, in most regions out of Africa, whether within the Tropics, as in the Antilles, or out of them, as at Gibraltar, is gradually exterminated."

The attention of the reader is next directed to the influence of foreign tropical climates upon negroes, and the degree of mortality of these people in the United States. From the extensive and reliable statistics brought forward by our author, it appears that while the blacks in the United States have increased tenfold, those of the British West Indies have diminished in the proportion of five to two. Of the whole 1,700,000 and their progeny, but 660,000 remained at the date of emancipation. Since this epoch the diminution in their numbers has been going on still more rapidly. According to Dr. Dowler,* the blacks imported from Africa, everywhere beyond the limits of the Slave States of North America, tend to extinction. The Liberian experiment, the most favorable ever made, is no exception to this general statement. During thirty-two years the number of colored persons sent to Liberia amounted to 7592, which number was reduced to 6 or 7,000 at the end of that time. The black race is evidently doomed to extinction in the West Indies. At the end of the first quarter of the present century Humboldt estimated their number at 1,960,000. Mr. Macgregor, in 1847, gives the total aggregate at 1,300,000; showing a decline in the preceding quarter of a century, of 660,000. Humboldt says that the whole Archipelago of the West Indies, which now comprises 2,400,000, negroes and mulattoes, free and slaves, received from 1670 to 1825, nearly 5,000,000

* New Orleans Medical and Surgical Journal, September, 1856.

Africans, and he assures us that the slaves would have diminished since 1820, with great rapidity, but for the fraudulent continuation of the slave trade.

Another interesting topic discussed by our author is, the relative susceptibility of various pure and hybrid races to yellow and other fevers. Negroes are nearly exempt from yellow fever. The susceptibility of mulattoes is in direct proportion to the infusion of white blood. Mexicans and Hispano-Mexicans are infinitely more liable to yellow fever than mulattoes of any grade. According to Dr. Blair* the liability of the white races to yellow fever, as compared with the dark, is as 8·436 per cent., to ·00004. At Vera Cruz, as well as in the West Indies, the Coolies are exempt from it, while in Cuba, the Chinese are much less amenable to it than Europeans. Pure-blooded Mexicans suffer more from its effects than pure-blooded Spaniards or mixed breeds. It is also asserted that the cross-breeds of negroes and Mexicans are liable to this disease, just in proportion to the amount of blood of the latter race, contained in their veins. This holds good with the cross-breeds of whites and negroes. With few exceptions, the affinity of yellow fever for the races of men is in direct proportion to the lightness of complexion. According to Clot-Bey the reverse of this is true of the plague. "Thus in all the epidemics, the negro race suffers most; after these, the Berbers or Nubians; then the Arabs of Hedjáz and Yemen; then the Europeans; and among these especially the Maltese, Greeks, and Turks, and generally the inhabitants of South Europe."† From the ample statistics collected by Dr. La Roche, it appears that the highest rate of mortality from yellow fever, is almost invariably among the pure white races—as the Germans, Anglo-Saxons, &c.

But we can no longer follow our author through the valuable statistics which he has industriously gathered from various sources, and it must be said, cogently and learnedly used, in support of the diversity doctrine. We cannot take leave of his excellent paper, however, without presenting the following conclusions, to which he has arrived:

"1. That the earth is naturally divided into zoological realms—each possessing a climate, Fauna, and Flora, exclusively its own.

"2. That the Fauna of each realm originated in that realm, and that it has no consanguinity with other Faunas.

"3. That each realm possesses a group of human races, which, though not identical in physical and intellectual characters, are closely allied with one another, and are disconnected from all other races. We may cite, as examples, the white races of Europe, the Mongols of Asia, the blacks of Africa, and the aborigines of America.

---

* Report on the first eighteen months of the fourth Yellow Fever Epidemic of British Guiana. See Brit. & For. Med.-Chir. Review, Jan. and April, 1845.

† De la Peste, 1840, p. 7, and Coup d'Œil sur la Peste, 1851.

"4. That the types of man, belonging to these realms, antedate all human records, by thousands of years; and are as ancient as the Faunas of which each forms an original element.

"5. That the types of man are separated by specific characters, as well marked and as permanent as those which designate the species of other genera.

"6. That the climates of the earth may be divided into PHYSICAL and MEDICAL; and that each species of man, having its own physiological and pathological laws, is peculiarly affected by both climates.

"7. That no race of man can be regarded as cosmopolite; but that those races which are indigenous to latitudes intermediate between the equator and poles, approach nearer to cosmopolitism than those of the Arctic or the Torrid Zone.

"8. That the assertion, that any one race ever has, or ever can be, assimilated to all physical or all medical climates, is an hypothesis unsustained by a single historical fact, and opposed to the teachings of natural history."

The turning of a leaf confronts us next with Mr. Gliddon, who at the commencement of Chapter V, throws down the gauntlet of controversy, and proceeds at once to do battle in behalf of his peculiar "polygenistic" views. With the limits assigned us already overrun, we cannot venture, how much soever we might wish, to enter upon an analysis of this and the succeeding chapters, comprising as they do, more than one-third of the entire work. This is the less to be regretted, however, since Mr. Gliddon has been so long before the public as a writer and a lecturer, that his Ethnological views are already too well known to require any exposition in this place. It will be sufficient, therefore, briefly to say, that starting with an introductory citation from the Cosmos of Humboldt, he proceeds to carry the reader over the whole ground of dispute between those who contend for the unity, and those who maintain the diversity of human origin. Then follows a lengthy inquiry into the antiquity of man, viewed in its historical, chronological, and palæontological aspects. In the course of this inquiry Mr. Gliddon attempts to show that in America, humatile men, and humatile monkeys occupy the same palæontological zones; that whilst all such remains of man are exclusively of the American Indian type, the monkeys, called Hapale, Cebus, Callithrix, &c., are equally "terræ geniti" of this continent; and that the types or special forms both of men and monkeys, are permanent and indestructible. Finally, we are presented in Chapter VI, with a commentary upon the principal distinctions observable among the various groups of humanity, and a short essay upon the geographical distribution of the Simiæ in relation to that of some inferior types of men.

It will thus be seen, that in noticing this elaborate and truly important work, we have wielded the pen analytical rather than the pen critical. Regarding the volume as at once the most recent and most complete exponent of Ethnological science in 1857, we have sought, in justice to its authors and our readers, to present a correct account of its scope and character, rather than to single out and dwell upon its merits and defects. Its chief merit lies, not so much in the various conclusions towards which it points, as in the immense array of facts which have been gathered together from the most varied and numerous sources. From beginning to end it is characterized by great labor and research. Especially is this true of the contributions of Mr. Gliddon, whose enormous reading gives to his chapters the appearance of a comprehensive commentary upon the entire range of both the scientific and biblical literature of Ethnology. Indeed, he appears to have rendered himself thoroughly acquainted with all the Ethnological books, pamphlets, papers, reviews, discussions, &c., that have been published, while his immense correspondence with both European and American Ethnologists makes him acquainted with new facts and discoveries long before they are published to the world. Of this fact the reader will find several instances in the work under notice. To the practical student of Ethnology, therefore, the work recommends itself as an indispensable index or book of reference; to the popular reader it is a valuable exponent of the present condition and tendencies of the science; while to the historian and the statesman it offers food for the deepest reflection.

The mechanical execution of the volume reflects the highest credit upon the liberality, taste, and enterprise of its publishers. Clear, bold type, superfine paper, nine admirable lithographs, some of which are beautifully colored, one hundred and ninety woodcuts, a magnificent tinted lithographic tableau containing fifty-four human portraits, and another large tinted map showing the geographical distribution of monkeys, and certain inferor types of men, are among the attractive features, which show that the spirited publishers have, indeed, "spared neither exertion nor outlay to make the book itself worthy of the patronage bestowed upon it." No better evidence of the extent and value of this patronage can be given, than the long list of subscribers appended to the work, in which we notice the names of many who at present adorn the walks of literature and science.

